# P068: Rapid detection of imipenem-resistant Acinetobacter baumannii for the surveillance culture using mass spectrometry

**DOI:** 10.1186/2047-2994-2-S1-P68

**Published:** 2013-06-20

**Authors:** D-K Kang, H Lee, S-I Joo, E-C Kim

**Affiliations:** 1 Infection Control Office, Seoul National University Hospital, Seoul, Korea, Republic Of; 2 Department of Laboratory Medicine, Seoul National University Hospital, Seoul, Korea, Republic Of

## Introduction

Korean Antimicrobial Resistance Monitoring System reported in 2010 that the resistance rate of *Acinetobacter baumannii* against imipenem was 71.7%. Increasing requirement of surveillance for Imipenem-resistant *Acinetobacter baumannii* (IRAB), specifically designed to hospital-acquired infection control activities. It needs to detect IRAB more rapidly to prevent further spread on healthcare facilities.

## Methods

Total 69 active surveillance samples were tested, which consist of 51 endotracheal aspirates, 15 sputum and three nasopharyngeal swabs. The respiratory samples for the surveillance of IRAB were inoculated into 2 mL of MacConkey broth containing 10 µg/mL of imipenem and incubated overnight at 37°C. For the rapid identification of IRAB, total 69 pellets obtained after the centrifugation of 1.5 mL were applied on mass spectrometry. The results of the mass spectrometry were compared to those of standard culture using MacConkey agar and VITEK2.

## Results

29 IRAB and 32 IRAB were identified by mass spectrometry and conventional sub-culturing MacConkey agar, respectively. Compared to the conventional subculture on MacConkey agar, the sensitivity and specificity of mass spectrometry method were 90.6% and 100%, respectively. Total 40 IRAB-negative samples by mass spectrometry were one *A. nosocomialis*, two *A. junii*, one *Staphylococcus aureus*, three *Enterococcus faecium*, one *Stenotrophomonas maltophilia*, one *Enterobacter aerogenes* and 31 no-reliables.

## Conclusion

The IRAB were detected two days earlier by mass spectrometry, compared to conventional sub-culturing MacConkey agar. Rapid detection of IRAB for surveillance culture using mass spectrometry must be a useful method for screening and improving infection control strategies aimed at limiting the spread of IRAB and shortening the period of isolation.

## Disclosure of interest

None declared

